# Dynamics of lung-infiltrating virus-specific T cells associated with age-dependent SARS-CoV-2 pneumonia severity

**DOI:** 10.1371/journal.ppat.1013866

**Published:** 2026-01-14

**Authors:** Rise Kurokawa, Chatherine Silas Mtali, Innocent John Daniel, Thorbjorg Einarsdottir, Omnia Reda, Nobuko Irie, Wajihah Sakhor, Koki Niimura, Mitsuyoshi Takatori, Kenji Sugata, Chanidapa Adele Tye, Yorifumi Satou, Masahiro Ono, Takushi Nomura

**Affiliations:** 1 Division of Virology and Pathology, Joint Research Center for Human Retrovirus Infection, Kumamoto University, Kumamoto, Japan; 2 Division of Genomics and Transcriptomics, Joint Research Center for Human Retrovirus Infection, Kumamoto University, Kumamoto, Japan; 3 Collaboration Unit for Infection, Joint Research Center for Human Retrovirus Infection, Kumamoto University, Kumamoto, Japan; 4 Department of Life Sciences, Imperial College London, London, United Kingdom; 5 AIDS Research Center, National Institute of Infectious Diseases, Japan Institute for Health Security, Tokyo, Japan; Washington University School of Medicine in Saint Louis: Washington University in St Louis School of Medicine, UNITED STATES OF AMERICA

## Abstract

Coronavirus disease 2019 (COVID-19) pneumonia is prevalent in the elderly infected with severe acute respiratory syndrome coronavirus 2 (SARS-CoV-2); however, the mechanisms underlying its age-dependent pathogenesis remain unclear. In this study, we established a mouse-adapted SARS-CoV-2 strain infected Nr4a3-Tocky mouse model to examine T-cell dynamics associated with disease severity. Nr4a3-Tocky mice allow the analysis of the dynamics and induction of antigen-reactive T cells following antigen recognition *in* *vivo* using fluorescent Timer protein. SARS-CoV-2-infected adult mice exhibited transient body weight loss and recovery, whereas aged mice developed severe pneumonia. BALF viral RNA was comparable between 1–4 days post-inoculation (d.p.i.), but declined in adults at 5 d.p.i. Aged mice displayed stronger inflammation as indicated by scRNA-seq, and higher levels of inflammatory cytokines (TNF-α, CCL2, CXCL10 and IL-6) in BALF correlated with weight loss. Timer analysis revealed induction of antigen-reactive T cells in the adult lungs at 5 and 8 d.p.i., which inversely correlated with disease severity. Additionally, S-specific IFN-γ ⁺ CD8 ⁺ T cells were detected at 5 d.p.i. in adults, whereas detection of antigen-specific T cells was delayed in aged mice. These results suggest that the coexistence of age-related lung inflammation and delayed induction of antigen-specific T cells is linked to more severe pneumonia, while earlier T-cell responses are associated with improved viral control and milder disease. In this study, we utilized a novel mouse model enabling characterization of antigen-reactive T cells in the local tissue, and investigated inflammatory responses in the lung together with lung-infiltrating virus-specific T cells, finding the dynamics of these immunological parameters associated with the age of the mice. Our analysis provides new insights into understanding how age-related T-cell dysfunction is associated with the severity of SARS-CoV-2 pneumonia.

## Author summary

COVID-19 pneumonia is prevalent in the elderly infected with SARS-CoV-2; however, its age-dependent pathogenesis is unclear. We established a SARS-CoV-2-infected Nr4a3-Tocky mouse model, which enables analysis of the dynamics and responses of antigen-reactive T cells, and identified age-dependent disease phenotypes. SARS-CoV-2-infected adult mice exhibited transient body weight loss and recovery, whereas aged mice developed severe pneumonia. SARS-CoV-2-infected adult mice induced T-cell responses in the lungs five days after infection, and showed reduced viral RNA levels in the respiratory tract. In aged mice, induction of T-cell responses was delayed in the context of the stronger inflammatory environment in the lung, and SARS-CoV-2 pneumonia was severe. Across these infected animals, the coexistence of age-related lung inflammation and delayed induction of antigen-specific T cells is linked to more severe pneumonia, while earlier T-cell responses are associated with improved viral control and milder disease. In conclusion, our multilayered analysis of the lung immunological state provides new insights to understanding how age-related T-cell dysfunction is associated with the severity of SARS-CoV-2 pneumonia.

## Introduction

Although the COVID-19 pandemic has been controlled by the development of effective vaccines, the virus remains a significant threat to people with underlying health conditions such as obesity, diabetes, and elderly people with such a condition have a significant risk for developing severe COVID-19 [1–4]. While previous studies have revealed the mechanisms behind increased risk of severe disease by COVID-19 caused by underlying disease, the mechanisms for age-related pathogenesis have yet to be identified. Aging modifies immune function and increases the risk of infections developing into severe disease. The T-cell populations responsible for cellular immunity maintain their reactivity and immunological memory to exogenous antigens by complex mechanisms, highly affected by aging [5–7]. Older individuals have attenuated induction of humoral/cellular immune responses induced by vaccinations, indicating a deterioration in the function of memory development of adaptive immunity with aging [8–10]. Studies on COVID-19 in humans have demonstrated that a decrease in naïve T-cell fraction in aged individuals correlates with disease severity [11,12]. Pulmonary vascular endotheliitis and thrombi develop in the lungs in SARS-CoV-2 infection, resulting in diffuse alveolar damage [13,14]. Tissue damage could be caused by viral replication, excessive inflammation by induced inflammatory cytokines or chemokines. Reduction or dysfunction of regulatory T cells (Tregs), which suppress excessive activation of immune responses, is potentially associated with severe pneumonia in SARS-CoV-2 infection [15,16].

Excessive T-cell activation in lung tissue mediates severe pneumonia in patients with SARS-CoV-2 infection [17–21]. Notably, the analysis of single-cell RNA-seq datasets [22] for bronchoalveolar lavage fluids from patients with moderate and severe COVID-19 showed altered CD4^+^ T-cell differentiation; atypically activated T-cells with high CD25 expression were increased while the expression of the Treg-specific transcription factor Foxp3 was reduced in patients with severe SARS-CoV-2 infection [16]. This finding suggests a role of Treg in moderating severe lung inflammation in SARS-CoV-2 patients and supports the need to further investigate mechanisms controlled by T-cells in the lung during the early phase of SARS-CoV-2 infection. Reduced T-cell receptor (TCR) diversity, impaired TCR signaling pathway, and loss of naïve T-cell fraction are associated with age-related dysfunction of T cells [5,23–27], and further analysis of the relationship between T-cell aging and the mechanism of severe COVID-19 is required. SARS-CoV-2 infected lungs present coexisting environments, where tissue damage by viral replication, exogenous and endogenous antigen production, and tissue inflammation occur; thus, the influence of immunopathology can be significant. Hence, the direct analysis of T-cell response dynamics in the lungs, rather than peripheral blood, in the acute or subacute phases of SARS-CoV-2 infection is vital to decipher how severe pneumonia develops. Because analyzing the human lung in the acute or subacute phase of SARS-CoV-2 infection is challenging, analysis of T-cell response dynamics in the lung parenchyma using animal models of SARS-CoV-2 infection is essential for understanding the pathogenesis of severe lung inflammation. The Timer-of-cell-kinetics-and-activity (Tocky) system enables analysis of temporal changes in immune cells *in vivo* during activation and differentiation using fluorescent Timer protein transgenic reporter [28]. Using the Nr4a3 gene, which is downstream of the TCR signaling, Nr4a3-Tocky mice allow analysis of the temporal dynamics of antigen-reactive T cells following antigen recognition.

Animal models including nonhuman primates [29–31], hamsters [32–34], and mice [35–38], have been used for studying SARS-CoV-2 infection. Among these, mice are the most useful for mechanistic studies due to the availability of various transgenic strains. There have been limitations in the use of C57BL/6 background mice in SARS-CoV-2 studies, because of limited susceptibility to infection. Nonetheless, some field isolate variants [39,40] and artificially established mouse-adapted virus strains [36–38] are pathogenic to C57BL/6 background mice and are suitable for infection experiments. In this study, we established an animal model of SARS-CoV-2 infection using Nr4a3-Tocky mice in a C57BL/6J background with the mouse-adapted SARS-CoV-2/QHmusX strain [36,37], which recapitulates age-dependent differences in disease progression. Because immunosenescence involves many alterations in immune function, we investigated whether age-related changes in the induction of antigen-specific T-cell responses are linked to the severity of SARS-CoV-2 pneumonia. By examining lung-infiltrating virus-specific T cells using Nr4a3-Tocky mice during SARS-CoV-2 infection, we identified T-cell dynamics associated with severe pneumonia.

## Results

### Age-dependent pathogenicity of SARS-CoV-2 infection in Nr4a3-Tocky mice

**Fig 1:**
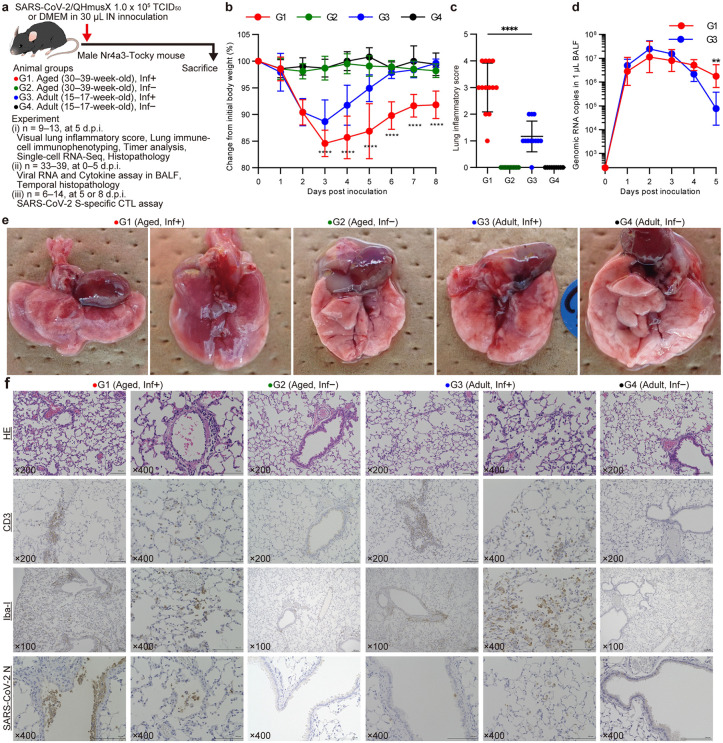
Experimental infection of Nr4a3-Tocky mice with SARS-CoV-2/QHmusX. At least fifteen Nr4a3-Tocky mice of different ages (in weeks) in each group were intranasally inoculated with 1.0 × 10^5^ TCID_50_/30 μL of SARS-CoV-2/QHmusX per mouse or with medium for the mock-infected groups. The body weight of mice was measured daily, and changes from the initial day of inoculation were calculated. **(a)** Illustration of the inoculation procedure applied to Nr4a3-Tocky mice across two age groups. **(b)** Comparative data on body weight changes post SARS-CoV-2 infection, demonstrating more significant weight loss in aged mice. ****P < 0.0001 indicates comparisons between G1 and G3 by Mann–Whitney *U* test. **(c)** Lung inflammatory scores based on visual evaluation at 5 d.p.i., illustrating severe pneumonia in aged mice and milder effects in adult mice. The comparison between G1 and G3 was performed using Mann–Whitney *U* test. **(d)** SARS-CoV-2 viral RNA levels in BALF post infection in aged and adult animals. Day 0 data represent pre-challenge baseline values. Viral RNA levels below 2,500 copies per 1 μL of BALF were considered below the limit of detection. **P < 0.005 indicates comparisons by Mann–Whitney *U* test. **(e)** Photographs showing lung appearance at 5 d.p.i., with notable differences between aged and adult mice. Photo credit: Takushi Nomura, Kumamoto University. **(f)** Representative lung sections at 5 d.p.i. were subjected to hematoxylin and eosin (H&E) staining and immunohistochemical analysis for CD3, Iba-1, and SARS-CoV-2 nucleocapsid (N) protein.

We addressed the age-dependent effects of SARS-CoV-2 infection on pneumonia severity using Nr4a3-Tocky mice, which were categorized into aged (30–39 weeks old) and adult (15–17 weeks old) groups (G1 aged infected, G2 aged control, G3 adult infected, G4 adult control, **Fig 1A**). Both groups were intranasally inoculated with the SARS-CoV-2/QHmusX strain. Interestingly, while all mice except one aged mouse survived the viral challenge, distinct differences were observed in their recovery patterns. The adult group experienced a transient body weight loss, recovering after 4 days post-inoculation (d.p.i.). In contrast, the average body weight of the aged group showed significantly delayed recovery compared to the adult group (**Fig 1B**). One aged mouse reached the humane endpoint due to severe body weight loss at 5 d.p.i. and was euthanized following bronchoalveolar lavage fluid (BALF) collection. The change in body weight after 4 d.p.i. differed by animal in the aged group (**Figs 1B** and **S1A**). Severe pneumonia developed in the aged group, but not in the adult group. This was quantitatively supported by visual lung inflammatory scoring that correlated positively with the body weight loss at 5 d.p.i. (**Figs 1C** and **S1B**). Temporal analyses of SARS-CoV-2 viral RNA in BALF (**Fig 1D**) indicated no differences between aged and adult infected mice from 1 to 4 d.p.i. However, adult mice exhibited a decline in viral RNA after 4 d.p.i., whereas aged mice showed a more gradual decrease. Consequently, viral RNA levels at 5 d.p.i. were significantly higher in aged infected mice. Histopathological analyses of lung tissues revealed age-dependent differences in inflammatory responses over the course of infection. Aged infected mice exhibited alveolar wall thickening as early as 2 d.p.i., whereas inflammatory changes in adult infected mice appeared from 3 d.p.i. (**S2** and **S3 Figs**). Subsequently, adult mice tended to recover from inflammatory changes gradually after 4 d.p.i., whereas aged mice maintained continuous inflammation, although complete destruction of alveolar structures was not detected in the observation period. At 5 d.p.i., extensive pulmonary inflammation and edema were noted in the aged group (**Fig 1E**), with histological analyses confirming alveolar wall thickening and cellular exfoliation (**Figs 1F** and **S4**). However, the adult group displayed only minor changes, indicative of a milder inflammatory response (**Figs 1F** and **S4**). Further immunohistochemical analyses demonstrated aggregation of CD3 ⁺ T cells and Iba-1 ⁺ macrophages around blood vessels in both groups at 3–4 d.p.i. At 5 d.p.i., aged and adult groups showed similar patterns of CD3^+^ T cell and Iba-1^+^ macrophage presence in the alveoli, suggesting a robust immune cell infiltration in both groups (**S2** and **S3 Figs**). Additionally, SARS-CoV-2 infected cells, as identified by viral N protein⁺ cells, were observed in bronchial epithelial cells of both groups at 1–2 d.p.i. (**S2** and **S3 Figs**). However, the distribution of SARS-CoV-2 infected cells differed significantly between the groups from 3 d.p.i.; aged mice exhibited these cells in both bronchioles and alveoli, while viral N protein^+^ cells were confined to the alveolar spaces in adult mice (**Figs 1F** and **S4**). These results collectively suggest that aging significantly modulates the severity and dynamics of pulmonary inflammation and viral clearance in SARS-CoV-2 infection.

### Inflammatory cell infiltration and T-cell dynamics in SARS-CoV-2 infection in Nr4a3-Tocky mice

**Fig 2:**
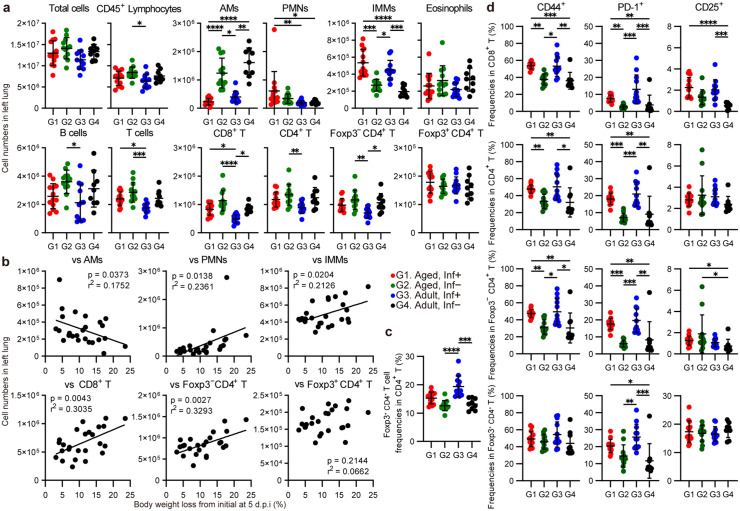
Cell populations and T-cell activation marker expressions in the lung post-SARS-CoV-2 infection at 5 d.p.i. Lung-derived cells were isolated from the left lung lobes at 5 d.p.i. and subjected to flow cytometric analysis for cell fractionation analysis. **(a)** Cell numbers of alveolar macrophages (AMs), polymorphonuclear cells (PMNs), inflammatory monocyte macrophages (IMMs), eosinophils, lymphocytes, B cells, CD8^+^ T cells, Foxp3^−^ CD4^+^ T cells, and Foxp3^+^ CD4^+^ T cells in CD45^+^ MNCs in lung tissue, demonstrating the effect of SARS-CoV-2 infection. **(b)** Correlations between the cell numbers in the left lung and the ratio of body weight loss at 5 d.p.i. from initial time in infected mice. **(c)** Frequencies of Foxp3^+^ CD4^+^ T cells in CD4^+^ T cells, indicating elevation of Foxp3^+^ CD4^+^ T-cell frequencies in the infected adult group. **(d)** Frequencies of CD44^+^, PD-1^+^, and CD25^+^ cells in CD8^+^ T cells, CD4^+^ T cells, Foxp3^−^ CD4^+^ T cells, and Foxp3^+^ CD4^+^ T cells, demonstrating similar T-cell activation in infected aged and adult mice.

To understand the inflammatory response within the lungs during SARS-CoV-2 infection, we analyzed infiltrating immune cells in the lung isolated from the left lobe of Nr4a3-Tocky mice at 5 d.p.i. (**Fig 2A**). Interestingly, the cell numbers of alveolar macrophages (AMs) were significantly reduced in both aged and adult groups following infection, compared to mock-infected controls, with no notable differences between the aged and adult groups infected with SARS-CoV-2. In contrast, inflammatory monocyte macrophages (IMMs) were significantly increased within the infected cohorts, although this increase did not vary significantly between aged and adult mice. The cell numbers of polymorphonuclear leukocytes (PMNs) were higher in the infected aged group than the infected adult group, while eosinophils remained unchanged. The cell numbers of CD8^+^ T cells were lower in the infected adult group. There was no significant difference in the cell numbers of CD4^+^ T cells between infected groups and their respective uninfected groups. Cell numbers from the left lung of infected mice were correlated with body weight loss at 5 d.p.i., revealing an inverse correlation with AM, whereas positive correlations with PMN, IMM, CD8^+^ T, and Foxp3^−^ CD4^+^ T cells (**Fig 2B**). The frequency of Foxp3^+^ CD4^+^ T cells within CD4^+^ T cells tended to be greater in the infected adult group compared to the uninfected groups, but was not significantly different between infected groups (**Fig 2C**). In further characterizing the immune response, we analyzed T-cell activation in the lungs of Nr4a3-Tocky mice infected with SARS-CoV-2. Frequencies of CD44^+^, PD-1^+^, and CD25^+^ cells in CD8^+^ T, CD4^+^ T-cell populations are detailed in **Fig 2D**. The frequency of CD44^+^ cells, indicative of migration to peripheral tissues, was elevated in the infected groups across CD8^+^ T, CD4^+^ T and Foxp3^−^ CD4^+^ T cells, but not in Foxp3^+^ CD4^+^ T cells. The examination of PD-1^+^ T cells tended to show higher frequencies in infected animals across all T-cell subsets. The frequencies of CD25^+^ cells, which are associated with T-cell activation, demonstrated fewer changes in the infected mice than the frequencies of CD44^+^ or PD-1^+^ cells, except in CD8^+^ T cells. In summary, animals infected with SARS-CoV-2 displayed an enhanced T-cell activation profile compared to their mock-infected counterparts.

### Antigen-reactive T-cell dynamics in Nr4a3-Tocky mice with SARS-CoV-2 infection

**Fig 3:**
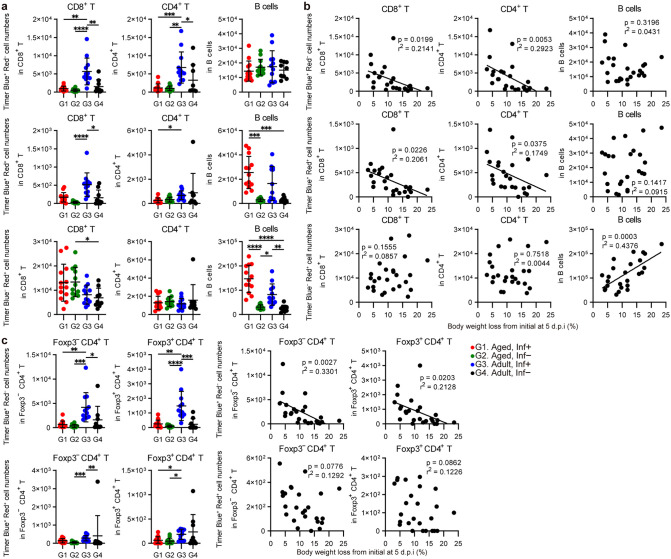
Timer analysis of lung-derived T/B cell populations in SARS-CoV-2-infected mice at 5 d.p.i. **(a)** The flow cytometric analysis results of fluorescent Timer proteins in left lung-derived cells, highlighting the cell numbers of antigen-reactive T and B cells in aged and adult mice infected with SARS-CoV-2 compared to mock-infected controls at 5 d.p.i. Timer^+^ cells are classified into three subsets: Timer blue^+^ red^−^, indicating recent newly antigen presentation stimulus; Timer blue^+^ red^+^ indicating continuous antigen presentation stimulus; and Timer blue^−^ red^+^, indicating previously arrested antigen presentation stimulus. **(b)** The inverse correlation between the cell numbers of Timer blue^+^ red^−^, Timer blue^+^ red^+^ and Timer blue^−^ red^+^ antigen-reactive T cells in the lungs and disease severity in SARS-CoV-2-infected mice. **(c)** The flow cytometric analysis results of fluorescent Timer proteins in subpopulations of CD4^+^ T cells and the inverse correlation between the numbers of antigen-reactive Foxp3^−^ CD4^+^ T cell and Foxp3^+^ CD4^+^ T cell, and disease severity.

Using Nr4a3-Tocky mice, the maturation of Timer protein can be used to decipher the dynamics of antigen recognition of T cells and B cells. In the current analysis, Timer^+^ cells were classified into three subsets: Timer blue^+^ red^−^, indicating recent newly antigen recognition; Timer blue^+^ red^+^ indicating continuous antigen recognition; and Timer blue^−^ red^+^, indicating previous antigen recognition. Flow cytometric analysis results of fluorescent Timer proteins in lung-derived cells from infected mice at 5 d.p.i. are displayed in **Fig 3**. Adult infected mice induced significantly higher cell numbers of Timer blue^+^ red^−^ antigen-reactive T cells in both CD8^+^ T and CD4^+^ T cell populations compared with the infected aged group and mock-infected groups (**Fig 3A**). Conversely, infected aged animals did not induce significant Timer blue^+^ red^−^ or Timer blue^+^ red^+^ antigen-reactive CD8^+^ and CD4^+^ T-cells. Both infected groups tended to show higher cell numbers of Timer blue^+^ red^+^ and Timer blue^−^ red^+^ antigen-reactive B cells compared to the mock-infected groups. The low cell numbers of Timer blue^+^ red^+^ antigen-reactive T cells in infected groups suggested that antigen-reactive T cells are short-lived in immunopathological environments. Cell numbers of Timer blue^+^ red^−^ and Timer blue^+^ red^+^ antigen-reactive CD8^+^ and CD4^+^ T cells in the lungs were inversely correlated with body weight loss, whereas Timer blue^−^ red^+^ B cells were positively correlated with disease severity (**Fig 3B**). Timer analysis of the CD4^+^ T cell subsets, Foxp3^−^ CD4^+^ T cell and Foxp3^+^ CD4^+^ T cell, revealed that infected adult mice induced a higher number of Timer blue^+^ red^−^ antigen-reactive T cells, and the induced reactive T cells were inversely correlated with disease severity (**Fig 3C**). The cell number of Timer blue^+^ cells, which combines Timer blue^+^ red^−^ and Timer blue^+^ red^+^ antigen-reactive T cells, in each T-cell subset was significantly higher in the infected adult mice, and the cell number of Timer blue^+^ T cells in each T cell subset was inversely correlated with body weight loss (**S5A** and **S5B Fig**). The cell number of Timer blue^+^ antigen-reactive B cells appeared to be elevated in both the infected aged and adult groups (**S5A Fig**). Furthermore, the numbers of Timer blue^+^ antigen-reactive T cells were significantly correlated with the Foxp3^+^ CD4^+^ T-cell frequencies in CD4^+^ T cells (**S5C Fig**), suggesting a connection between the induction of antigen-reactive T cells and expansion of the Foxp3^+^ CD4^+^ T-cell population in the lung in SARS-CoV-2 infection.

### SARS-CoV-2 S-specific CD8^+^ T-cell dynamics after infection

**Fig 4:**
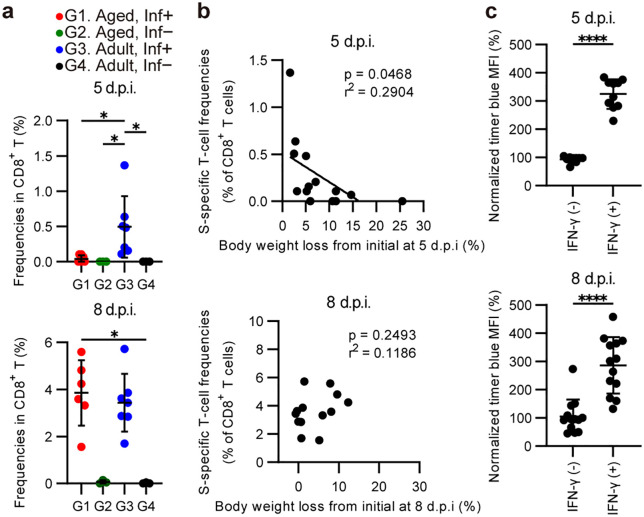
SARS-CoV-2 S-specific T-cell response analysis of lung-derived cells in SARS-CoV-2-infected mice. **(a)** Comparisons of SARS-CoV-2 S-specific CD8^+^ T-cell frequencies in lung-derived cells at 5 or 8 d.p.i. in aged and adult mice. **(b)** The correlation between the SARS-CoV-2 S-specific CD8^+^ T-cell frequencies and disease severity in SARS-CoV-2-infected mice. **(c)** Comparison of Timer blue mean fluorescence intensity (MFI) between IFN-γ⁺ and IFN-γ ⁻ CD8 ⁺ T-cell subsets after SARS-CoV-2 S overlapping peptide stimulation in infected mice that showed an S-specific CD8 ⁺ T-cell response at 5 or 8 d.p.i. The MFI of Timer blue in total CD8 ⁺ T cells from unstimulated control samples was normalized to 100, and relative MFI values for IFN-γ⁺ and IFN-γ⁻ subsets following S-specific stimulation are shown.

SARS-CoV-2 spike (S) protein-specific CD8 ⁺ T-cell responses in the lung were analyzed at 5 and 8 d.p.i. using stimulation with overlapping peptides spanning the S protein and intracellular IFN-γ staining of CD8 ⁺ T cells. At 5 d.p.i., infected mice exhibited higher frequencies of IFN-γ ⁺ S-specific CD8 ⁺ T cells compared to uninfected controls (**S6 Fig**). Among the infected groups, adult mice showed significantly higher frequencies of S-specific CD8 ⁺ T cells than the aged mice (**Fig 4A**). In infected mice at 5 d.p.i., the frequency of S-specific CD8 ⁺ T cells was inversely correlated with body weight loss (**Fig 4B**). These findings are consistent with results from Timer-based analysis and suggest that adult mice have a greater capacity to induce virus antigen-specific T-cell responses than aged mice. At 8 d.p.i., infected mice exhibited further increases in the frequency of IFN-γ ⁺ S-specific CD8 ⁺ T cells, indicating progressive antigen-specific T-cell induction (**S6 Fig**). At this time point (8 d.p.i.), both adult and aged infected mice displayed comparable levels of S-specific CD8 ⁺ T-cell responses (**Fig 4A**). No correlation was observed between the frequency of S-specific CD8 ⁺ T cells and body weight loss in infected mice at 8 d.p.i. (**Fig 4B**). At 5 d.p.i., these results paralleled the Timer analysis, suggesting that adult mice initiate antigen-specific T-cell responses earlier after infection than aged mice, including the induction of SARS-CoV-2 S-specific CD8 ⁺ T cells. SARS-CoV-2 S-specific IFN-γ ⁺ CD8 ⁺ T cells showed markedly higher Timer blue intensity (**Fig 4C**), indicating that antigen stimulation-induced Timer protein expression is associated with IFN-γ production in Nr4a3-Tocky mice during SARS-CoV-2 infection.

### Examination of T-cell functions contributing to the regulation of inflammation in the lung by scRNA-seq analysis

**Fig 5:**
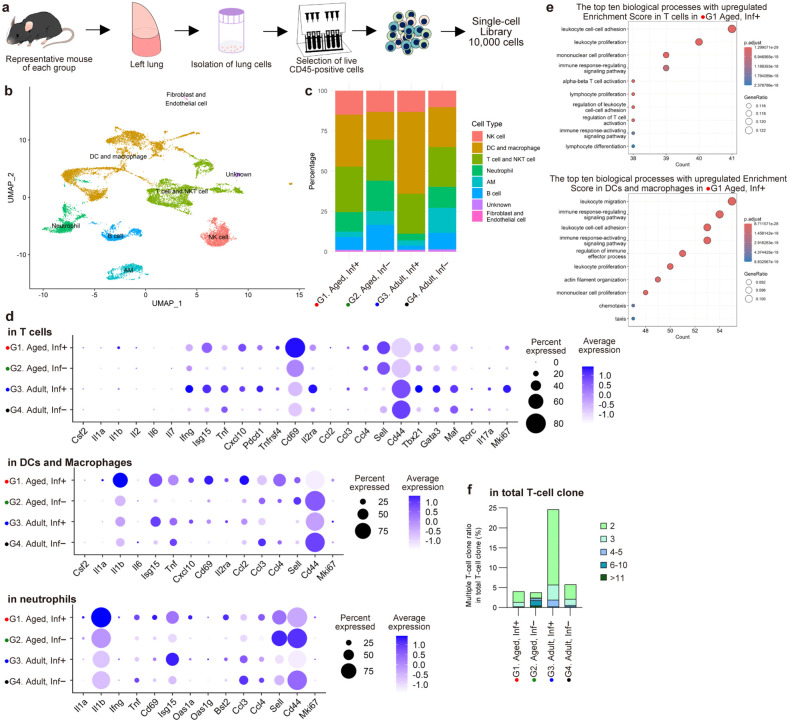
Single-cell gene expression analysis using lung derived cells from representative mice of each group at 5 d.p.i. **(a)** Illustration of experimental design of single-cell gene expression analysis. Lung derived cells were isolated from the left lung of a representative mouse in each group by enzyme dissociation methods. CD45^+^ viable cells from lung were purified by using magnetic beads selections for dead cell removal and CD45 positive selection. Approximately 10,000 cells per sample were loaded onto the Chromium controller (10x Genomics). **(b)** UMAP plot of merged and harmonized scRNA-seq data (G1-4), with each of the 8 clusters labeled. Each dot represents one of 15,641 cells (n = 4). **(c)** Stacking plot showing relative frequencies of cell populations in each mouse. **(d)** Dot plots showing expression patterns of selected genes in each mouse T cells, dendritic cells (DCs) and macrophages, and neutrophils. Color denotes expression level, dot size denotes percentage of cells in each cluster expressing a given gene. **(e)** Gene Ontology (GO) enrichment analysis of differentially expressed genes based on biological processes. Genes showing a significantly higher expression in T cells (325 genes), and DCs and macrophages (526 genes) in an aged infected animal (G1) were subjected to GO enrichment analysis. **(f)** Bar graph showing multiple T-cell clone ratio in total T-cell clones in TCR repertoire analysis.

Single-cell gene expression analysis was performed at 5 d.p.i. on viable CD45^+^ lung cells from representative mice of each group to evaluate T-cell functions potentially associated with excessive/attenuated lung inflammation (**Fig 5A**). UMAP plots and clustering by marker genes (**S1 Table** and **S7A Fig**) were conducted for G1-G4, revealing distinct populations of NK cells, dendritic cells (DCs) and macrophages, neutrophils, T cells and NKT cells, alveolar macrophages, and B cells (**Figs 5B**, **5C**, and **S7B**). NKT cell cluster was annotated by sub-clustering of T cells and NKT cells in G1-G4 mice (**S7C Fig**) by marker genes (**S2 Table**). In SARS-CoV-2-infected mice in G1-G4, the frequency of DCs and macrophages was higher compared to uninfected control mice, while the frequency of alveolar macrophages was lower (**Fig 5C**), consistent with the results from the flow cytometric analysis. Dot plots and violin plots showed gene expression trends in each cell population in G1-G4 mice (**Figs 5D** and **S7D**). In infected mice, T cells exhibited high gene expression of activation markers such as Cxcl10, Pdcd1, Tnfrsf4, and Il2ra. In infected mice, DCs and macrophages highly expressed inflammatory cytokines, activation markers, and migration-related genes, including Il1b, Isg15, Tnf, Cxcl10, Cd69, Il2ra, Ccl2, Ccl3, and Ccl4. In aged infected mouse, the expression of inflammatory cytokines, including type I IFNs, activation markers, and migration-related genes was higher in DCs and macrophages compared to adult infected mouse, indicating stronger induction of inflammation (**Figs 5D**, **5E** and **S7D**). Neutrophils in the aged infected mouse also showed elevated expression of Il1b (**Figs 5D** and **S7D**). GO enrichment analysis of biological process in T cells from G1 and G3 revealed that genes associated with adhesion and activation were enriched in the aged infected mouse. (**Fig 5E**). Although flow cytometric analysis did not reveal significant differences in the frequency of T cells expressing activation markers between infected groups, single-cell analysis showed greater activation in T cells in aged infected mouse. TCR clonotype analysis indicated that multiple clones were more frequent in adult infected mouse, suggesting the proliferation of reactive T cells in the adult infected mouse (**Fig 5F**).

### Analysis of cytokine production in BALF associated with age-dependent severity of SARS-CoV-2 infection

**Fig 6:**
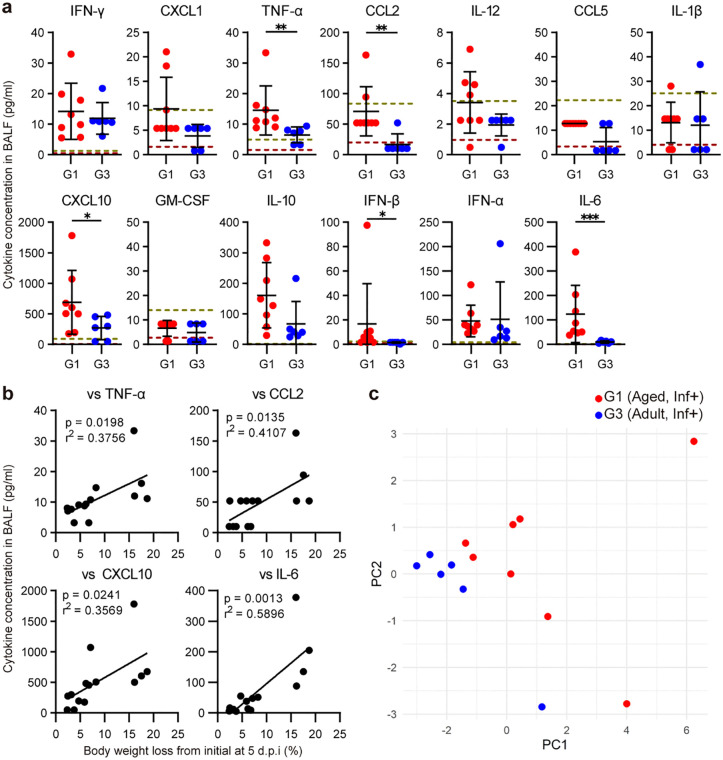
Cytokine quantification in BALF from SARS-CoV-2 in aged and adult mice at 5 d.p.i. **(a)** Cytokine concentrations in BALF collected at 5 d.p.i., from adult and aged mice infected with SARS-CoV-2. Horizontal reference lines mark the assay LOQ (gray-yellow dashed) and LOD (gray-red dashed). **(b)** Correlation analysis between cytokine concentrations in BALF and body weight loss at 5 d.p.i. **(c)** PCA of 13 BALF cytokines in adult and aged mice infected with SARS-CoV-2. PC1 (51.8%) and PC2 (16.6%) of the variance are shown on the axes.

To investigate the contribution of cytokine production to age-dependent SARS-CoV-2 disease severity, we quantified cytokine concentrations associated with virus infection in the BALF of adult and aged mice at 5 d.p.i. using a multiplex bead-based assay. The concentrations of cytokines, including TNF-α, CCL2, CXCL10, IFN-β, and IL-6 were significantly higher in aged mice compared to adult mice (**Fig 6A**). These tendencies of cytokine induction were consistent with our scRNA-seq data, suggesting higher expression of proinflammatory cytokines in the lungs of aged mice after SARS-CoV-2 infection. Furthermore, concentrations of TNF-α, CCL2, CXCL10, and IL-6 in BALF positively correlated with body-weight loss at 5 d.p.i. (**Fig 6B**), suggesting that enhanced inflammatory cytokine responses are associated with more severe disease in infected mice. Principal component analysis (PCA) of 13 BALF cytokine concentrations revealed a separation between adult and aged infected mice (**Fig 6C**), indicating age-dependent differences in the overall cytokine profile.

## Discussion

In this study, we established a SARS-CoV-2 infection animal model with age-dependent severity using Nr4a3-Tocky mice. Unlike the lethal infection model utilizing BALB/c mice [36,38], this model on a C57BL/6 genetic background permits detailed analysis of immune responses in the subacute phase of lung infection. Both aged and adult SARS-CoV-2-infected Nr4a3-Tocky mice exhibited comparable levels of inflammatory cell infiltration and T-cell activation in the lungs by flow cytometric analysis, indicating a similar potential for pneumonia development. However, while severe pneumonia was observed in aged mice, adult mice exhibited limited pneumonia, suggesting that age-related factors in adult mice may suppress pneumonia caused by SARS-CoV-2 infection, or other factors in aged mice may enhance inflammation. The elevated expression of inflammatory cytokine genes revealed by the single-cell RNA-seq data in DCs, macrophages, and neutrophils was observed in aged infected mouse, compared to adult infected mouse. Consistent with this, aged infected mice exhibited higher proinflammatory cytokine production in BALF than adult mice. An augmented myeloid-derived cytokine response was associated with more severe pneumonia in aged mice and may be linked to disease exacerbation. This research highlights the effectiveness of utilizing C57BL/6 transgenic mice as a model for SARS-CoV-2 infection, paving the way for more comprehensive studies into the pathogenesis of severe SARS-CoV-2 infection using a variety of transgenic mouse models.

Next, this study demonstrated that the induction dynamics of antigen-reactive and antigen-specific T-cell responses during the acute and subacute phases of lung infection are associated with the severity of SARS-CoV-2 pneumonia. It is plausible that the induction of antigen-specific T cells, which play a central role in viral control, may support pneumonia control. We hypothesized before this project that in severe cases, massive antigen presentation due to viral replication and tissue destruction in the lungs could promote uncontrolled T-cell activation, thereby potentially worsening pneumonia. The Nr4a3-Tocky mice we used in this study uniquely enable the investigation of antigen-reactive T-cell responses by Timer protein analysis [28]. The analysis of Timer protein in lung T cells of SARS-CoV-2-infected Nr4a3-Tocky mice revealed significant differences in antigen recognition dynamics between adult and aged mice. Adult infected mice exhibited higher cell numbers of recent newly induced antigen-reactive CD8^+^ T cells, Foxp3^−^ CD4^+^ T cells, and Foxp3^+^ CD4^+^ T cells compared to aged infected mice. These antigen-reactive T cells likely include both SARS-CoV-2-specific T cells and specific T cells cross-reacting with autoantigens from inflamed host lung tissue, suggesting that the highly induced T-cell response in adult mice is consistent with more effective viral control. It is also conceivable that a less severe pulmonary pathology resulting from SARS-CoV-2 infection could lead to the induction of antigen-specific T-cell responses. It was not possible to identify the precise epitope antigens recognized by T cells using Timer fluorescent analysis in Nr4a3-Tocky mice. In this study, we conducted single-cell TCR repertoire analysis of lung-derived cells to examine the expansion of T-cell clones in SARS-CoV-2-infected lung. The TCR repertoire data indicates that T-cell clonal expansion in the adult lung is consistent with the antigen-reactive T-cell response dynamics identified through Timer protein analysis. Additionally, we sequenced the VDJ regions of individual T-cell clones; however, identifying the epitope antigens recognized by TCRs remains challenging with currently available technology. We anticipate that development of antigen recognition prediction technology will enable the identification of antigens recognized by T cells induced in the subacute phase of viral infections in the future. Using a conventional antigen-specific CD8 ⁺ T-cell assay by stimulation with overlapping peptides spanning the S protein and intracellular IFN-γ staining of CD8 ⁺ T cells, we found that adult infected mice had already induced S-specific CD8 ⁺ T cells at 5 d.p.i., whereas aged mice failed. At 8 d.p.i., aged mice also induced S-specific CD8 ⁺ T-cell responses, reaching frequencies comparable to adults. Thus, while adult mice can induce virus-specific CD8 ⁺ T cells earlier after infection, aged mice display delayed induction, which may be consistent with impaired control of pneumonia. Accordingly, age-dependent differences in the timing of virus-specific CD8 ⁺ T-cell induction were associated with disease severity. Although the SARS-CoV-2 S protein represents only part of the viral antigenic repertoire, these temporal differences in S-specific CD8 ⁺ T-cell induction are consistent with and help to contextualize the findings from the Timer analysis. Temporal analyses in influenza virus infected mouse models show that virus-specific CD8 ⁺ T cells become detectable in the lung by ~5–6 d.p.i. and typically peak around 7–10 d.p.i. [41–44]. In SARS-CoV-2 mouse studies, pronounced lung CD8 ⁺ T-cell responses are often reported by ~7–8 d.p.i. [45,46]. Although our findings indicate that Timer blue^+^ antigen-reactive T cells and S-specific IFN-γ ⁺ CD8 ⁺ T cells are detectable in the lung as early as 5 d.p.i. in adult animals, the response is delayed in aged animals. These data align with prior work in respiratory virus models and extend the concept of age-associated delays in virus-specific CD8 ⁺ T-cell priming and induction to the SARS-CoV-2 infection model. In aged mice, immunosenescence likely impairs speedy induction of antigen-specific T-cell responses, which may be linked to disease severity. Single-cell gene expression analysis of the lungs revealed that T cells in aged infected mouse exhibited increased expression of genes associated with adhesion and activation. These findings suggest that adult infected mice may be in a state more conducive to inducing organized T-cell responses. This study provides integrative evidence for the impact of aging on the T-cell immune system and its effect on pathogenesis in SARS-CoV-2 infection. It is generally known that aging impairs the induction of T-cell responses induced by vaccination [8,9,47]. Furthermore, age-related decline in antigen-specific responses to pathogens during infection has been reported [48,49], with potential contributing factors including decreased antigen presentation capacity by APCs [50,51], reduced TCR diversity [23,24,52], and aging of TCR signaling [26,27]. Studies of COVID-19 in humans have indicated that a decrease in naïve T-cell fraction correlates with disease severity [11,12]. Indeed, this study observed a decline in the frequency of antigen-specific T cells and a reduction in reactive clones in aged infected mice, suggesting that defective antigen-specific T-cell responses were linked to exacerbated pneumonia. There are reports of impaired antibody induction following vaccination in the elderly [8,53,54], and like T cells, B cells also decline in function with age. However, a secondary finding of this study was that there was no difference in the cell number of antigen-reactive B cells in the lungs of adult and aged infected mice. This suggests that B-cell antigen recognition is less affected by aging, though the precise mechanisms remain unclear.

This study has several limitations. Our experimental results are correlative and do not establish a definitive causal mechanism linking delayed T-cell responses to disease exacerbation. The analysis of antigen-specific T-cell induction dynamics was limited to the lungs and was not evaluated in the upper respiratory tract or other lymphoid tissues. Therefore, our conclusions should be interpreted within the scope of lung T-cell responses and pneumonia. Finally, because we did not completely distinguish resident lung T cells from intravascular leukocytes, the spatial resolution of the lung T-cell compartment is limited. Further research with higher resolution analyses across compartments will advance these observations and investigate the causal relationships underlying disease pathogenesis.

Because T-cell responses play a central role in viral control, early induction of antigen-reactive and antigen-specific T cells in adult mice may be associated with attenuated pneumonia. BALF viral RNA copies were comparable between adult and aged infected mice up to 4 d.p.i., indicating no age-related difference in viral replication during the acute phase. At 5 d.p.i., viral RNA levels diverged, with adults showing lower viral RNA copies consistent with more efficient clearance, potentially supported by earlier induction of virus-specific T-cell responses. Conversely, the heightened inflammatory milieu in aged mice with more severe pneumonia may impair proper induction of antigen-specific T cells. Immunosenescence is multifactorial and likely shapes both antiviral immunity and the degree of inflammation. Thus, dissecting the contribution and mechanisms of each component is important. Within this framework, our data identify an age-dependent delay in the induction of antigen-specific T cells as one age-dependent feature associated with severe pneumonia. Our findings support an age-dependent model in which the pulmonary inflammatory milieu and the timing of antigen-specific T-cell priming jointly shape disease outcomes in SARS-CoV-2 pneumonia, providing insights for future work on immunosenescence and infectious diseases.

## Methods

### Animal experiments

#### Ethics statement.

All animal experiments were conducted in strict adherence to welfare guidelines and were approved by the Animal Care and Use Committee at Kumamoto University, Kumamoto, Japan. This includes experiments involving transgenic mice, which were specifically approved by the Committee for Type 2 Use of Living Modified Organisms at the same institution. All procedures were in line with Japanese laws and regulations concerning animal welfare. In addition, all animal experiments were performed in a controlled environment within a biosafety level three laboratory at the Joint Research Center for Human Retrovirus Infection, ensuring compliance with the highest safety standards.

### Cells

VeroE6/TMPRSS2 cells [55] were provided by the Japanese Collection of Research Bioresources Cell Bank (National Institute of Biomedical Innovation, Health and Nutrition, Osaka, Japan). VeroE6/TMPRSS2 cells were cultured in Dulbecco’s modified Eagle’s medium (DMEM), low glucose (Thermo Fisher Scientific, Waltham, MA, USA, cat. # 11885084), 10% fetal bovine serum (FBS) (Cytiva, Tokyo, Japan, cat. # A8412-100ML), and antibiotics (100 U/mL penicillin and 0.1 mg/mL streptomycin) (Thermo Fisher Scientific, cat. # 15140122). Cells were maintained in 1.0 mg/mL geneticin (Thermo Fisher Scientific, cat. # 10131035) and used for analysis without geneticin.

### Virus stocks

VeroE6/TMPRSS2 cells were inoculated with the mouse-passaged SARS-CoV-2 QHmusX strain (GenBank Accession No.: LC605054) [36] (passage number 2) and cultured in DMEM supplemented with 2% FBS and antibiotics for 24 h. Culture supernatants containing propagated virus (passage number 3) were stored at −80°C. The infectivity titers of SARS-CoV-2 were analyzed using VeroE6/TMPRSS2 cells cultured in DMEM containing 2% FBS and antibiotics. Viral infectivity titers were expressed as 50% tissue culture infectious dose (TCID_50_)/mL, calculated according to the Reed–Muench method by analyzing the cytopathic effect.

### Mice

Nr4a3-Tocky is a BAC reporter transgenic mouse in a C57BL/6J background for the Nr4a3 gene using the FT Fast mCherry mutant as a reporter, as previously described [28]. All the Nr4a3-Tocky mice used in this study were homozygous for Nr4a3-Tocky and hemizygous or homozygous for Foxp3-IRES-EGFP. Wild-type C57BL/6J mice were purchased from Japan SLC (Shizuoka, Japan).

### Animal study design

Male Nr4a3-Tocky mice were used in the experiments, categorized into two age groups with reference to previous infectious disease mouse studies [38,56,57]: 15–17 weeks (Adult) and 30–39 weeks (Aged). Nr4a3-Tocky mice were anesthetized by intraperitoneal injection of 0.1 mL/10 g mouse body weight of domitor (0.03 mg/mL) (Nippon Zenyaku Kogyo Co., Ltd., Tokyo, Japan), vetorphale (0.50 mg/mL) (Meiji Animal Health Co., Ltd., Kumamoto, Japan), and midazolam (0.40 mg/mL) (Sandoz K.K., Tokyo, Japan) mixture. Animals were intranasally inoculated with 1.0 × 10^5^ TCID_50_ (30 µL) of mouse-passaged SARS-CoV-2 strain QHmusX. Mock-infected mice were inoculated with DMEM containing 2% FBS. Body weight was measured daily, and animals were euthanized under anesthesia for sample collection and subsequent analyses. Each group included a total of 15–65 mice. (i) Mice were euthanized at day 5 post-infection (5 d.p.i.) for visual assessment of lung inflammatory score based on gross lung appearance. Regions with clearly altered coloration were defined as pneumonic areas. A six-point scale (0–5) was applied according to the estimated proportion of affected lung tissue: 0 (no visible lesion), 1 (0–20%), 2 (20–40%), 3 (40–60%), 4 (60–80%), and 5 (>80%). The left lung was collected for viable immune cell isolation, followed by immunophenotyping, Timer analysis, and single-cell RNA-seq. The right lung was fixed in paraformaldehyde (PFA) in phosphate buffer (Fujifilm, Tokyo, Japan, cat. # 163–20145) and subjected to histopathological analysis (n = 9–13 per group). (ii) Mice were sacrificed at 0 d.p.i. to 5 d.p.i. for temporal analysis. Bronchoalveolar lavage fluid (BALF) was obtained by instilling 1 ml of PBS (Thermo Fisher Scientific, cat. # 10010023) through the trachea, followed by PFA fixation of the lungs for histological examination (n = 33–39 per group). (iii) To assess SARS-CoV-2-specific CTL responses by using overlapping peptide pool stimulation, viable cells were isolated from both lungs at 5 d.p.i. or 8 d.p.i. (n = 6–14 per group). The humane endpoint of infection experiment was set as the appearance of clinical signs of respiratory distress and >25% weight loss. C57BL/6 uninfected mice were used as controls for Timer analysis.

### Cell isolation from lung tissues

The lungs were dissociated with a gentleMACS Octo Dissociator with Heaters (Miltenyi, Bergisch Gladbach, North Rhine–Westphalia, Germany, cat. # 130-096-427) and the Lung Dissociation Kit for mouse (Miltenyi, cat. # 130-095-927) without perfusion. We shortened the heating time of the enzymatic treatment for the dissociation process from 30 min to 10 min to protect the Timer protein, whereas other procedures were performed according to the manufacturer’s protocol. The number of lung-derived cells obtained was counted microscopically.

### Surface marker profiling and Timer analysis in lung immune cells

Lung-derived cells were subjected to flow cytometric analysis using two patterns of surface staining. First, cells were washed and blocked with 1 μg anti-CD16/anti-CD32 (clone 93; Thermo Fisher Scientific, cat. # 14-0161-82) at 4°C for 15 min. Dead cells were stained and gated out with the LIVE/DEAD Fixable Near-IR Dead Cell Stain Kit (Thermo Fisher Scientific, cat. # L10119) in both staining patterns. Cells for population and Timer analysis in B cells in the lung (2.0 × 10^6^ cells/sample) were stained with the following antibodies at 4°C for 30 min: BUV395 anti-mouse CD45 (clone 30-F11; BD, Franklin Lakes, New Jersey, USA, cat. # 564279) at 1:400 dilution; PE-Cy7 anti-mouse CD3e (clone 145-2C11; BioLegend, cat. # 100320) at 1:100 dilution; BUV737 anti-mouse B220 (clone RA3-6B2; BD, cat. # 612838) at 1:200 dilution; BUV805 anti-mouse CD4 (clone RM4.5; BD, cat. # 741912) at 1:400 dilution; Alexa 700 anti-mouse CD8 (clone 53-6.7; BioLegend, cat. # 100730) at 1:200 dilution; BV785 anti-mouse CD11b (clone M1/70; BioLegend, cat. # 101243) at 1:200 dilution; BV711 anti-mouse Ly6G (clone 1A8; BioLegend, cat. # 127643) at 1:100 dilution; Alexa Fluor 647 anti-mouse Siglec-F (clone S17007L; BioLegend, cat. # 155520) at 1:100 dilution; PerCP-Cy5.5 anti-mouse Ly-6C (clone HK1.4; BioLegend, cat. # 128012) at 1:100 dilution. Cells were washed and fixed with paraformaldehyde in phosphate buffer. The gating strategy is shown in **S8 Fig**. Cell fractions were defined as described in a previous report [58]. Cells for the analysis of Timer protein in T cells and T-cell activation in the lung were stained with the following antibodies at 4°C for 30 min: PE-Cy7 anti-mouse CD3e (clone 145-2C11; BioLegend, cat. # 100320) at 1:100 dilution; BUV737 anti-mouse TCRb (clone H57-597; BD, cat. # 612821) at 1:400 dilution; PerCP anti-mouse CD4 (clone RM4–5; BioLegend, cat. # 100538) at 1:400 dilution; BV785 anti-mouse CD8 (clone 53-6.7; BioLegend, cat. # 100749) at 1:200 dilution; APC anti-mouse CD44 (clone IM7; BioLegend, cat. # 103012) at 1:400 dilution; BUV395 anti-mouse PD-1 (clone J43; BD, cat. # 744549) at 1:100 dilution; Alexa 700 anti-mouse CD25 (clone PC61; BioLegend, cat. # 102024) at 1:100 dilution. The gating strategy is shown in **S9 Fig**. Stained cells were analyzed using BD FACS Symphony A3 with FACS Diva v9.1 (BD). Timer blue, excited with the violet laser, was analyzed with the 470/15 450LP filter; Timer red, excited with the yellow-green laser, was analyzed with the 610/20 600LP filter. Foxp3 transcript levels, reported by EGFP, were excited with a yellow-green laser and analyzed with the 586/15 570LP filter. Flow cytometric data were evaluated using FlowJo v10.7.1 (FlowJo LLC, Ashland, Oregon, USA).

### Single-cell RNA-seq library construction from enriched lung CD45^+^ cells

We conducted gene expression analysis at the single-cell level to analyze lung immune activation and TCR repertoire. To analyze immune activation in monocyte lineage cells and lymphocytes with the TCR repertoire, we constructed a single-cell RNA-seq library using the CD45^+^ cell fraction, derived from lung tissue and isolated from one representative animal from each group. Cells obtained from the left lung were processed through a two-step magnetic bead selection process to isolate viable CD45^+^ cells, following the manufacturer’s protocols. First, negative selection was applied to remove dead lung cells using a Dead cell removal kit (Miltenyi, cat. # 130-090-101). Next, the live CD45^+^ cell fraction was recovered by positive selection using CD45^+^ microbeads (Miltenyi, cat. # 130-052-301). The harvested cells were filtered twice with a 40 µm filter and counted under a microscope. Finally, cells were resuspended in 0.025% BSA (Sigma-Aldrich, St. Louis, Missouri, USA, cat. # 130-052-301) and PBS, and were processed using Chromium Controller (10x Genomics, Pleasanton, California, USA), following the manufacturer’s protocols. Single-cell suspensions were processed using the Chromium Next GEM Single Cell 5′ Reagent Kits v2 (10x Genomics, cat. # PN-1000265). Enrichment of V(D)J segments and construction of the single-cell TCR library were performed using the Chromium Single Cell Mouse TCR Amplification Kit (10x Genomics, #PN-1000254). Gene expression libraries were prepared according to the manufacturer’s protocol. cDNA libraries were sequenced on the NextSeq500 platform (Illumina, San Diego, California, USA) to reach a minimum depth of 50,000 reads/cell.

### Single-cell RNA sequencing data analysis

Raw sequencing reads from the gene expression libraries were processed using Cell Ranger v7.1.0, aligning reads to the mm10 build of the mouse genome. The resulting output file, which is a gene-cell barcode matrix, was imported into R(59) (v4.4.1) and analyzed using Seurat [60] (v4.4.0). We created Seurat object with “CreateSeuratObject” function (min.cells = 3, min.features = 200). The dataset was filtered to remove low-quality cell data based on three metrics: (1) the number of unique genes detected in each cell; (2) the total number of molecules detected within a cell; and (3) the percentage of reads that map to the mitochondrial genome. Filtering was conducted with different criteria for each sample and cells that met the criteria were subjected to downstream analysis. Each cell data was extracted by the following criteria; > 200 nFeature_RNA (all samples), < 7,000 nFeature_RNA (G1-1), < 6,000 nFeature_RNA (G2-1, G3-9, G4-8), > 200 nCount_RNA (all samples), < 50,000 nCount_RNA (G1-1), < 40,000 nCount_RNA (G2-1, G3-9), < 30,000 nCount_RNA (G4-8), and < 5% mitochondrial genes. Obtained data were processed by default setting of Seurat’s “NormalizeData”, “FindVariableFeatures”, “ScaleData”, and “RunPCA” functions. The batch effect resulting from the difference in analysis day was corrected with “RunHarmony” function [61]. The dimension reduction analysis was conducted by uniform manifold approximation and projection (UMAP) with 30 dimensions for visualization. Clusters were calculated using Seurat’s “FindClusters” function with default parameters. Each identified cluster was manually annotated by “FindAllMarkers” function from Seurat and cell subset-defining marker genes defined according to a previous report [62] with additional references (**S1 Table**). T cell and NKT cell cluster was subjected to additional dimension reduction analysis, and manually annotated by cell subset-defining marker genes (**S2 Table**).

### Visualization of gene expression patterns in mouse lung cell subsets

Expression patterns of selected markers in each murine lung dataset were visualized in dot plot and violin plot using the “DotPlot” function in Seurat v4.4.0 [60] in R-4.4.1 [59] and “ggplot” function in ggplot2 v3.5.1 in R-4.4.1 [59]. Differences in gene expression were analyzed by Dunn’s multiple comparison test or Mann–Whitney *U* test.

### Differential gene expression and pathway analysis

Differential expression testing between samples was performed with Seurat’s “FindMarkers” function. Differentially expressed genes were identified with an adj *p* value < 0.05. Gene Ontology (GO) enrichment analysis was conducted with the Bioconductor package clusterProfiler (v4.12.2) using the org.Mm.e.g.,db database.

### TCR-seq analysis

TCR sequences were assembled with the Cell Ranger V(D)J pipeline (version v7.1.0), leading to an output file that contained the identification of the CDR3 sequence and the rearranged TCR gene. Cells with an identified pair of TCRα and TCRβ clonotype were isolated, and the others were discarded. Multiple T-cell clone ratio in total T-cell clone was calculated manually by using isolated data.

### Histopathology and immunohistochemistry

The lung tissues fixed in PFA were embedded in paraffin and sectioned. The tissue sections were stained with hematoxylin and eosin (H&E). Antigen retrieval of tissue sections was performed by autoclaving at 121°C for 10 min for immunohistochemistry. For detecting CD3, rabbit anti-CD3 monoclonal antibody (clone 2GV6; Ventana, Tucson, Arizona, USA, cat. # 790–4341) was used prediluted overnight at 4°C. For detecting Iba-1, rabbit anti-Iba-1 polyclonal antibody (Wako, Osaka, Japan, cat. # 019–19741) was used overnight at 4°C. For SARS-CoV-2 antigen detection, an anti-SARS-CoV-2 N-specific rabbit monoclonal antibody (clone 019; Sino Biological, Beijing, China cat. # 40143-R019) was used overnight at 4°C. A diaminobenzidine chromogen kit (HISTOFINE MAX-PO Rabbit, Nichirei Biosciences, Tokyo, Japan, cat. # 414341) was used as a chromogen to visualize horseradish peroxidase. Nuclei were counterstained with Mayer’s hematoxylin. Histological and immunohistochemical staining was performed by Biopathology Institute Co., Ltd, Oita, Japan.

### Viral RNA quantification in BALF

Viral RNA was extracted from 200 μL of BALF using QIAamp Viral RNA Mini Kit (Qiagen, cat. # 52906) and quantified by real-time PCR using the QuantiTect Probe RT-PCR Kit (Qiagen, cat. # 204443) and LightCycler 96 (Roche). Standard curve was generated using positive control RNA (Nihon Gene Research Laboratories Inc., cat. # JP-NN2-PC). The detection limit of the assay was 2,500 copies/mL.

### Cytokine quantification in BALF

Cytokine concentrations in BALF supernatants were measured by flow cytometry-based multiplex immunoassay using LEGENDplex Mouse Anti-Virus Response Panel (BioLegend, cat. # 740622) according to the manufacturer’s protocol. After completing all assay steps, the multiplex beads were incubated for 1 hour in PBS (Thermo Fisher Scientific, cat. # 10010023) containing 0.5% Triton X-100 (Merck, cat. # X100-5ML) for viral inactivation, then washed and transferred to a BSL2 laboratory for flow cytometric analysis on BD FACS Symphony A3. Acquired FCS files were analyzed online using LEGENDplex Data Analysis Software Suite (BioLegend), and cytokine concentrations in BALF were determined. For plotting and nonparametric analyses, values below LOD were imputed as LOD/2, and values between LOD and LOQ as the midpoint (LOD + LOQ)/2.

### Analysis of SARS-CoV-2 S-specific T-cell responses

SARS-CoV-2 S-specific T-cell frequencies were determined by intracellular staining of IFN-γ following antigen-specific stimulation. Lung-derived cells (>0.5 × 10^6^ cells/sample) were subjected to 6 hours stimulation culture in the presence of Brefeldin A (BD GolgiPlug Protein Transport Inhibitor, BD, cat. # 555029) with overlapping peptides spanning the full-length SARS-CoV-2 spike (S) protein (0.5 μg/mL per peptide; JPT Peptide Technologies, cat. # PM-WCPV-S-1). Cells were washed and blocked with 1 μg anti-CD16/anti-CD32 (clone 93; Thermo Fisher Scientific, cat. # 14-0161-82) at 4°C for 15 min. Dead cells were stained and gated out with the LIVE/DEAD Fixable Near-IR Dead Cell Stain Kit (Thermo Fisher Scientific, cat. # L10119). Intracellular IFN-γ staining was performed using the Cytofix/Cytoperm Fixation/Permeabilization Kit (BD, cat. # 554714) and stained with BUV395 anti-mouse CD45 (clone 30-F11; BD, cat. # 564279) at 1:400 dilution; PE-Cy7 anti-mouse CD3e (clone 145-2C11; BioLegend, cat. # 100320) at 1:100 dilution; BUV737 anti-mouse B220 (clone RA3-6B2; BD, cat. # 612838) at 1:200 dilution; BUV805 anti-mouse CD4 (clone RM4.5; BD, cat. # 741912) at 1:400 dilution; Alexa 700 anti-mouse CD8 (clone 53-6.7; BioLegend, cat. # 100730) at 1:200 dilution; Alexa647 anti-mouse IFN-γ (clone XMG1.2; BioLegend, cat. # 505814) at 1:200 dilution. Cells were washed and fixed with paraformaldehyde in phosphate buffer. The gating strategy is shown in **S10 Fig**. Stained cells were analyzed using BD FACS Symphony A3 with FACS Diva v9.1 (BD). Flow cytometric data were evaluated using FlowJo v10.7.1 (FlowJo LLC). The frequencies of SARS-CoV-2 S-specific IFN-γ⁺ cells in CD8 ⁺ T cells were calculated by subtracting the frequencies of non-specific IFN-γ⁺ cells observed in unstimulated control from those in samples stimulated by SARS-CoV-2 S overlapping peptides.

### Statistical analysis

Data are presented as means ± SD. All statistical analyses except for scRNA-seq data analysis were conducted using GraphPad Prism 9.5.0 (GraphPad, San Diego, California, USA). Statistical analysis between two groups was performed using Mann–Whitney *U* test or between multiple groups using Dunn’s multiple comparison test. Correlation analyses were performed using Pearson’s correlation test. *P* values <0.05 were considered significant and are shown as follows: *P < 0.05, **P < 0.01, ***P < 0.001, ****P < 0.0001

### Language editing and translation support by AI

We used DeepL (DeepL SE) and ChatGPT (OpenAI) to assist with English language editing and translation of parts of the manuscript. The authors reviewed and edited the text and take full responsibility for the content.

## Supporting information

S1 Table The list of marker genes and associated references for cell clustering of single-cell gene expression analysis. (PDF)

S2 Table The list of marker genes and associated references for T cell and NKT cell clustering of single-cell gene expression analysis. (PDF)

S1 Fig Detailed progression results of SARS-CoV-2 infection in Nr4a3-Tocky mice. (a) Individual body weight changes post-inoculation, highlighting transient weight loss and recovery in adult mice. (b) The correlation between body weight loss at 5 d.p.i. and visual lung inflammatory score was significant.
(TIF)

S2 Fig Temporal histopathological analysis of the lung in SARS-CoV-2 infected aged mice. Representative lung sections collected from 0 to 5 d.p.i. following BALF collection were subjected to hematoxylin and eosin staining (H&E), and immunohistochemistry for CD3, Iba-1 and SARS-CoV-2 N protein. Day 0 sections represent pre-challenge baseline histology.
(TIF)

S3 Fig Temporal histopathological analysis of the lung in SARS-CoV-2 infected adult mice. (TIF)

S4 Fig Whole-right lobe images of representative lung sections from each group. Histological photos of the whole right lung lobe at 5 d.p.i. stained with H&E, and immunohistochemistry for CD3, Iba-1 and SARS-CoV-2 N protein from each group, corresponding to the samples presented in **Fig 1E**. Boxed areas indicate the regions shown at higher magnification in **Fig 1E**.
(TIF)

S5 Fig Timer blue^+^ T and B cell analysis in SARS-CoV-2-infected mice at 5 d.p.i. (a) Flow cytometric analysis results of Timer blue^+^ T and B cells in lung-derived cells, demonstrating significant induction of antigen-reactive T cells in adult mice. (b) Correlations between Timer blue^+^ T/B-cell numbers and body weight loss, indicating an association of antigen-reactive T-cell induction and SARS-CoV-2 mild disease. (c) Correlation between Timer blue^+^ cell number in Foxp3^+^ CD4^+^ T cell and Foxp3^+^ CD4^+^ T-cell frequencies in CD4^+^ T-cell population.
(TIF)

S6 Fig SARS-CoV-2 S-specific CD8 ⁺ T-cell responses in infected and uninfected mice. Frequencies of IFN-γ⁺ SARS-CoV-2 S-specific CD8 ⁺ T cells in the lung at 5 and 8 d.p.i. in infected mice (aged and adult) versus age-matched uninfected controls.
(TIF)

S7 Fig Single-cell clustering and inflammatory gene expression analysis in aged and adult mice at 5 d.p.i. (a) Feature plots of marker gene expression used for cell clustering. (b) UMAP plot of scRNA-seq data (split by G1-4), with each of the 8 clusters labeled. The number of cells in each mouse is as follows: G1 (aged infected, n = 3,530), G2 (aged control, n = 2,284), G3 (adult infected, n = 6,011), G4 (adult control, n = 3,816). (c) UMAP plot of scRNA-seq data from T cell and NKT cell cluster. (d) Violin plot showing selected gene expressions in each mouse in T cells, dendritic cells (DCs) and macrophages, and neutrophils. The *p* values were evaluated by Dunn’s multiple comparison test.
(TIF)

S8 Fig Gating strategies for cell population analysis in lung tissues and Timer analysis in B cells. Representative flow cytometry gating strategies for identifying monocyte lineage cells, lymphocytes, and Timer protein expression in B cells from aged mice infected with SARS-CoV-2 and aged mock-infected mice at 5 d.p.i. Cell fractions were defined as described in a previous report.
(TIF)

S9 Fig Gating strategies for Timer and activation marker analysis in T cells. Representative flow cytometry gating strategies for identifying Timer protein maturation in T-cell subpopulations in adult SARS-CoV-2-infected and mock-infected group at 5 d.p.i.
(TIF)

S10 Fig A gating strategy for SARS-CoV-2 S-specific CD8^+^ T-cell analysis. A representative flow cytometry gating strategy for detection of IFN-γ^+^ CD8^+^ T cells after overlapping peptide stimulation in a SARS-CoV-2-infected mouse.
(TIF)
