# Supplementary material for: Dynamics of lung-infiltrating virus-specific T cells associated with age-dependent SARS-CoV-2 pneumonia severity
Source: PLoS Pathog. 2026 Jan 14;22(1):e1013866. doi: 10.1371/journal.ppat.1013866 (PMC12829949; doi:10.1371/journal.ppat.1013866)
Supplement: S1 Table — (PDF) [file ppat.1013866.s001.pdf]

S1 Table: The list of marker genes and associated references for cell clustering of single-cell gene expression analysis.

No	Annotation	Lineage marker genes	Reference
1	NK cell	Ncr1, Klrb1c	Grégoire C., et al., Immunological Reviews, 2007(1)
2	Dendritic cell/Macrophage	Csf1r, Xcr1, Fscn1, Cd68, Itgam, Itgax	Zilionis R., et al., Immunity, 2019(2), Murray PJ., et al., Nature Reviews Immunology, 2011(3)
3	T cell and NKT cell	Cd3e, Il7r	Painter MW., et al., Journal of Immunology, 2011(4), Paget C., et al., Mucosal Immunology, 2013(5)
4	Neutrophil	S100a8, S100a9, Csf3r	Zilionis R., et al., Immunity, 2019(2)
5	Alveolar macrophage	Csf2rb, Mrc1	Suzuki T., et al., Nature, 2014(6)
6	B cell	Cd19, Cd79a	Carter RH., et al., Science, 1992(7), Zilionis R., et al., Immunity, 2019(2)
7	Unknown		
8	Fibroblast/Endothelial cell	Cldn5, Col1a2	Gillich A., et al., Nature, 2020(8), Angelidis I., et al., Nature Communications, 2019(9)

References

1. Gregoire C, Chasson L, Luci C, Tomasello E, Geissmann F, Vivier E, et al. The trafficking of natural killer cells. Immunol Rev. 2007;220(1):169-82.

2. Zilionis R, Engblom C, Pfirschke C, Savova V, Zemmour D, Saatcioglu HD, et al. Single-Cell Transcriptomics of Human and Mouse Lung Cancers Reveals Conserved Myeloid Populations across Individuals and Species. Immunity. 2019;50(5):1317-34 e10.

3. Murray PJ, Wynn TA. Protective and pathogenic functions of macrophage subsets. Nat Rev Immunol. 2011;11(11):723-37.

4. Painter MW, Davis S, Hardy RR, Mathis D, Benoist C, Immunological Genome Project C. Transcriptomes of the B and T lineages compared by multiplatform microarray profiling. J Immunol. 2011;186(5):3047-57.

5. Paget C, Trottein F. Role of type 1 natural killer T cells in pulmonary immunity. Mucosal Immunol. 2013;6(6):1054-67.

6. Suzuki T, Arumugam P, Sakagami T, Lachmann N, Chalk C, Sallese A, et al. Pulmonary macrophage transplantation therapy. Nature. 2014;514(7523):450-4.

7. Carter RH, Fearon DT. CD19: lowering the threshold for antigen receptor stimulation of B lymphocytes. Science. 1992;256(5053):105-7.

8. Gillich A, Zhang F, Farmer CG, Travaglini KJ, Tan SY, Gu M, et al. Capillary cell-type specialization in the alveolus. Nature. 2020;586(7831):785-9.

9. Angelidis I, Simon LM, Fernandez IE, Strunz M, Mayr CH, Greiffo FR, et al. An atlas of the aging lung mapped by single cell transcriptomics and deep tissue proteomics. Nat Commun. 2019;10(1):963.
