# Supplementary material for: Dynamics of lung-infiltrating virus-specific T cells associated with age-dependent SARS-CoV-2 pneumonia severity
Source: PLoS Pathog. 2026 Jan 14;22(1):e1013866. doi: 10.1371/journal.ppat.1013866 (PMC12829949; doi:10.1371/journal.ppat.1013866)
Supplement: S2 Table — (PDF) [file ppat.1013866.s002.pdf]

S2 Table: The list of marker genes and associated references for T cell and NKT cell clustering of single-cell gene expression analysis.

No	Annotation	Lineage marker genes	Reference
1	$CD3^{+}$ T	Cd3e, Il7r	Painter MW., et al., Journal of Immunology, 2011(1)
2	$CD4^{+}$ T	Cd4	
3	$CD8^{+}$ T	Cd8a	
4	NKT	Cd4,Cd8a, Klrb1c	Paget C., et al., Mucosal Immunology, 2013(2)

References

1. Painter MW, Davis S, Hardy RR, Mathis D, Benoist C, Immunological Genome Project C. Transcriptomes of the B and T lineages compared by multiplatform microarray profiling. J Immunol. 2011;186(5):3047-57.

2. Paget C, Trottein F. Role of type 1 natural killer T cells in pulmonary immunity. Mucosal Immunol. 2013;6(6):1054-67.
